# Systemic inflammation and pro-inflammatory cytokine profile predict response to checkpoint inhibitor treatment in NSCLC: a prospective study

**DOI:** 10.1038/s41598-021-90397-y

**Published:** 2021-05-25

**Authors:** Diego Kauffmann-Guerrero, Kathrin Kahnert, Rosemarie Kiefl, Laura Sellmer, Julia Walter, Jürgen Behr, Amanda Tufman

**Affiliations:** 1 Divison of Respiratory Medicine and Thoracic Oncology, Department of Internal Medicine V and Thoracic Oncology Centre Munich (TOM), University of Munich (LMU), Munich, Germany; 2 Comprehensive Pneumology Center Munich (CPC-M), Member of the German Center for Lung Research (DZL), Munich, Germany; 3 Department of Respiratory Medicine and Thoracic Oncology, Hospital of the University of Munich, Ziemssenstraße 1, 80336 Munich, Germany

**Keywords:** Non-small-cell lung cancer, Immunosurveillance, Cancer, Cytokines

## Abstract

Treatment with single agent immune checkpoint inhibitors (ICIs) has tremendously changed second line therapy in NSCLC. However, there are still no reliable biomarkers predicting response and survival in this group of patients. PD-L1 revealed to be a correlating, but no perfect marker. Therefore, we sought to investigate in this prospective study, whether inflammation status and cytokine profile could serve as additional biomarkers guiding treatment decision for single agent ICIs in NSCLC. 29 stage IV NSCLC patients receiving single agent PD-1 checkpoint-inhibitor in second line were prospectively enrolled. Inflammatory scores and cytokine profiles (IL-6, IL-8, IL-10, IFN-γ and TNFα) have been obtained before treatment and at the time of the first staging. Cytokine profiles were correlated with response and survival. Patients with signs of pre-therapeutic inflammation (elevated, NLR, SII, IL-6, IL-8) showed significantly lower response to ICI treatment and reduced PFS. Contrary, elevated levels of IFN-γ revealed to characterize a subgroup of patients, who significantly benefits from ICI treatment. Furthermore, low systemic inflammation and high levels of IFN-γ characterized patients with long term-response to ICI treatment. Pre-therapeutic assessment of inflammation and cytokine profiles has the ability to predict response and survival in NSCLC patients treated with single agent ICIs.

## Introduction

Non-small-cell lung cancer (NSCLC) accounts for about 85% of all lung cancer diagnoses and most patients are diagnosed in advanced stage needing systemic treatment. Immune checkpoint inhibitors (ICIs) have tremendously changed the treatment landscape of NSCLC during the last 5 years. Demonstrating prolonged progression free survival (PFS) and overall survival (OS) compared to docetaxel in second-line treatment, nivolumab (regardless PDL-1 expression) and pembrolizumab (in patients with a PD-L1 expression of at least 1%) have been approved in 2015 by the European Medicines Agency (EMA)^1, 2^. At present, several checkpoint inhibitors are approved in first- and further lines. However, little is known about biomarkers that reliably predict response and prognosis during the treatment with ICIs. High PD-L1 expression correlates with good response to immunotherapy, but is by far not a perfect marker.

First data indicate that signs of acute neutrophilic inflammation predict poor response to ICI therapy^3, 4^. However, the value of inflammatory cytokines in the immune response derived from ICI treatment is poorly understood. First results indicate that markers of acute inflammation such as IL-6 or IL-8 are associated with reduced response^5, 6^. Furthermore, markers of lymphocytic immune response as TNF-α, IFN-γ or IL-10 have be linked to better results under ICI treatment^6^. However, conclusive and especially prospective data on the impact of inflammation and cytokine profiles on the success of ICI therapy in NSCLC patients are missing.

In this study, we prospectively assessed pre-therapeutic and longitudinal inflammatory profiles (common laboratory values and cytokines) of advanced NSCLC patients treated with nivolumab or pembrolizumab monotherapy in second line.

## Patients and methods

### Study population

We prospectively enrolled stage IV patients diagnosed and treated with second line single agent PD-1 checkpoint inhibition at our tertiary care lung cancer center between 2016 and 2019. All patients received either nivolumab or pembrolizumab. Patients’ characteristics in terms of age, gender, performance status, tumor stage, smoking status, histology, ethnicity and PD-L1 expression were assessed at baseline. Age was calculated at the time of enrollment. All patients gave their informed consent to participate at the study. The study was approved by the local ethics board [ethical board of the university of Munich (LMU)] and conducted in accordance with the Declaration of Helsinki.

### Treatment and evaluation of tumor response

All patients received either nivolumab or pembrolizumab as second line treatment as approved in Germany. All patients received PET-CT or CT baseline staging before ICI treatment to confirm progression after first-line treatment and to exclude infectious or rheumatic diseases, which could lead to differences in cytokine profiles or survival. Treatment response was evaluated using CT or PET-CT based monitoring using the Response Evaluation Criteria in Solid Tumors 1.1 (RECIST, stable disease, partial or complete response and progressive disease). Patients with partial response or stable disease were classified as “responder”. Progression free survival (PFS) was calculated from the date of treatment start until the date of verified progressive disease in CT or PET-CT scan.

### Examined laboratory values and scores

Pre-therapeutic neutrophil-, lymphocyte- and platelet counts as well as pre-therapeutic CRP and albumin levels were obtained from laboratory measurements before initiation of treatment. Inflammation scores were calculated using the equations in Table 1.

**Table 1:** Equations for inflammatory scores.

	**Score**
**Neutrophil to lymphocyte ratio (NLR)**	
abs. neutrophil count/abs. lymphocyte count	
**Platelet to lymphocyte ratio (PLR)**	
abs. platelet count/abs. lymphocyte count	
**Systemic-inflammation-index (sii)**	
abs. platelet count x NLR	
**Modified Glasgow Prognostic Score (mGPS)**	
C-reactive protein ≤ 0.05 mg/dl and albumin ≥ 3.5 g/dl	0
C-reactive protein ≤ 0.05 mg/dl and albumin < 3.5 g/dl	1
C-reactive protein > 0.05 mg/dl and albumin ≥ 3.5 g/dl	1
C-reactive protein > 0.05 mg/dl and albumin < 3.5 g/dl	2

### Serum samples and cytokine measurement

Serum samples were obtained twice (before treatment start and at the time of the first staging). Blood samples were centrifuged, divided into aliquots and stored at − 20 °C. To measure cytokine concentration we used the “Human Cytokine-Inflammation (9-plex)” Kit by BioVendor. The samples were measured according the manufacturer guidelines.

### Statistical analysis

Metric data were displayed as means with standard deviations. PFS was compared using the Kaplan–Meier method with log-rank test. Categorical data were analyzed by the chi-square test. As some variables showed deviations from normal distribution according to the Kolmogorov–Smirnov-test, the Mann–Whitney-U-test as a non-parametric method was used to compare means of different groups. ROC analysis was used to calculate cut-off values. Multivariate analysis was performed using a logistic regression for binominal variable and a COX regression model for survival-data. A p value of < 0.05 was determined to be statistically significant. All statistical analyses were performed using SPSS statistical software (version 26, IBM Corp., Armonk, NY, USA).

### Ethical approval

The authors are accountable for all aspects of the work in ensuring that questions related to the accuracy or integrity of any part of the work are appropriately investigated and resolved.

## Results

### Study population

Between 2016 and 2019, we prospectively enrolled 29 stage IV NSCLC patients receiving single agent PD-1 checkpoint-inhibitor in second line. The study cohort was predominantly male (82.8%), the mean age was 64.1 years. Two thirds of patients were diagnosed with adenocarcinoma. PD-L1 expression was stratified into three groups (0%, 1–49% and ≥ 50%) with about one third of patients in each group. Most patients were active or former smokers and about two thirds received nivolumab therapy, whereas the remaining patients were treated with pembrolizumab.

Baseline characteristics of the study cohort are summarized in Table 2.

**Table 2:** Patients’s baseline characteristics.

	**n = 29**
**Gender**	
Male	24 (82.8%)
Female	5 (17.2%)
**Age**	64.1 ± 8.5
Male	64.4 ± 8.4
Female	62.6 ± 9.9
**Ethnicity**	
Caucasian	26 (89.7%)
Asian	0 (0%)
Other	3 (10.3%)
**Histology**	
Adenocarcinoma	19 (65.5%)
Squamous cell carcinoma	10 (34.5%)
**PD-L1 expression**	
0%	9 (31.0%)
1–49%	9 (31.0%)
≥ 50%	11 (37.9%)
**Performance status (ECOG)**	
0	6 (20.7%)
1	15 (51.7%)
2	8 (27.6%)
**Smoking status**	
Active	9 (31.0%)
Former	18 (62.1%)
Never	2 (6.9%)
**Treatment**	
Nivolumab	18 (62.1%)
Pembrolizumab	11 (37.9%)

Table 1 summarizes the baseline characteristics of the study cohort.

*ECOG* European Cooperative Oncology Group, *PD-L1* Programed death ligand 1.

### Pre-therapeutic inflammatory scores

First, we tested whether standard blood values indicating acute inflammation and scores calculated from these values correlate with response to immunotherapy or PFS.

**Figure 1:**
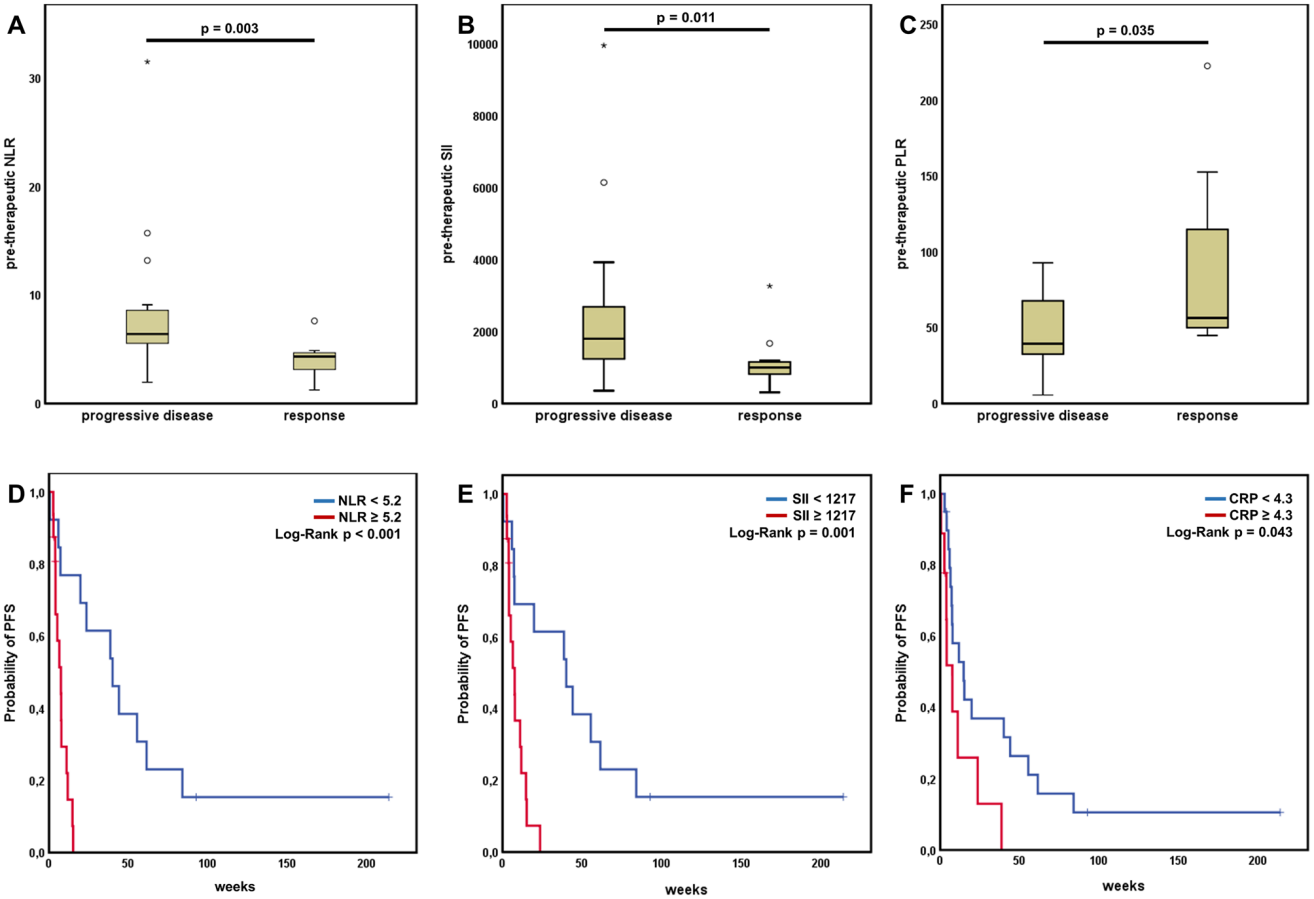
Pre-therapeutic inflammatory scores predict response and survival. Patients with initial response showed significantly lower levels of (**A**) neutrophil to lymphocyte ratio (NLR), (**B**) systemic inflammation index (SII) or elevated levels of (**C**) platelet to lymphocyte ratio (PLR). PFS was significantly longer in patients with lower NLR (**D**), lower SII (**E**) and lower CRP (**F**).

NLR was significantly elevated in patients with primary progressive disease (p = 0.003, Fig. 1A). We performed a ROC analysis to determine a NLR value of 5.2 to be the best cutoff for response in our cohort. Patients with NLR equal or greater than 5.2 showed significantly reduced PFS under checkpoint inhibitor treatment (median PFS 7.43 weeks [95% CI 3.37–11.49] vs. 40.0 weeks [95% CI 16.01–64.0], p < 0.001) (Fig. 1D).

SII was significantly increased in patients showing primary progression under immunotherapy (p = 0.011, Fig. 1B). The best cut-off value for SII regarding response was 1217. Patients with SII equal or higher than 1217 had significantly reduced PFS compared with patients harboring lower values (median PFS 7.51 weeks [95% CI 3.26–11.88] vs. 40.0 weeks [95% CI 11.48–68.52], p = 0.001) (Fig. 1E).

PLR was revealed to be significantly increased in patients responding to immunotherapy (p = 0.035, Fig. 1C). However, PLR levels did not show any impact on patients’ survival.

Patients responding on immunotherapy showed a trend to lower CRP levels, although this difference was not statistically significant. However, choosing the optimal cut-off (4.3 mg/dl) in our cohort, showed a significant PFS advantage in patients with low CRP values (median PFS 14.71 weeks [95% CI 4.18–25.25] vs. 7.57 weeks [95% CI 2.71–12.44], p = 0.043) (Fig. 1F).

mGPS did not discriminate between response and progressive disease or subgroups with better survival.

### Pre-therapeutic cytokine profile

**Figure 2:**
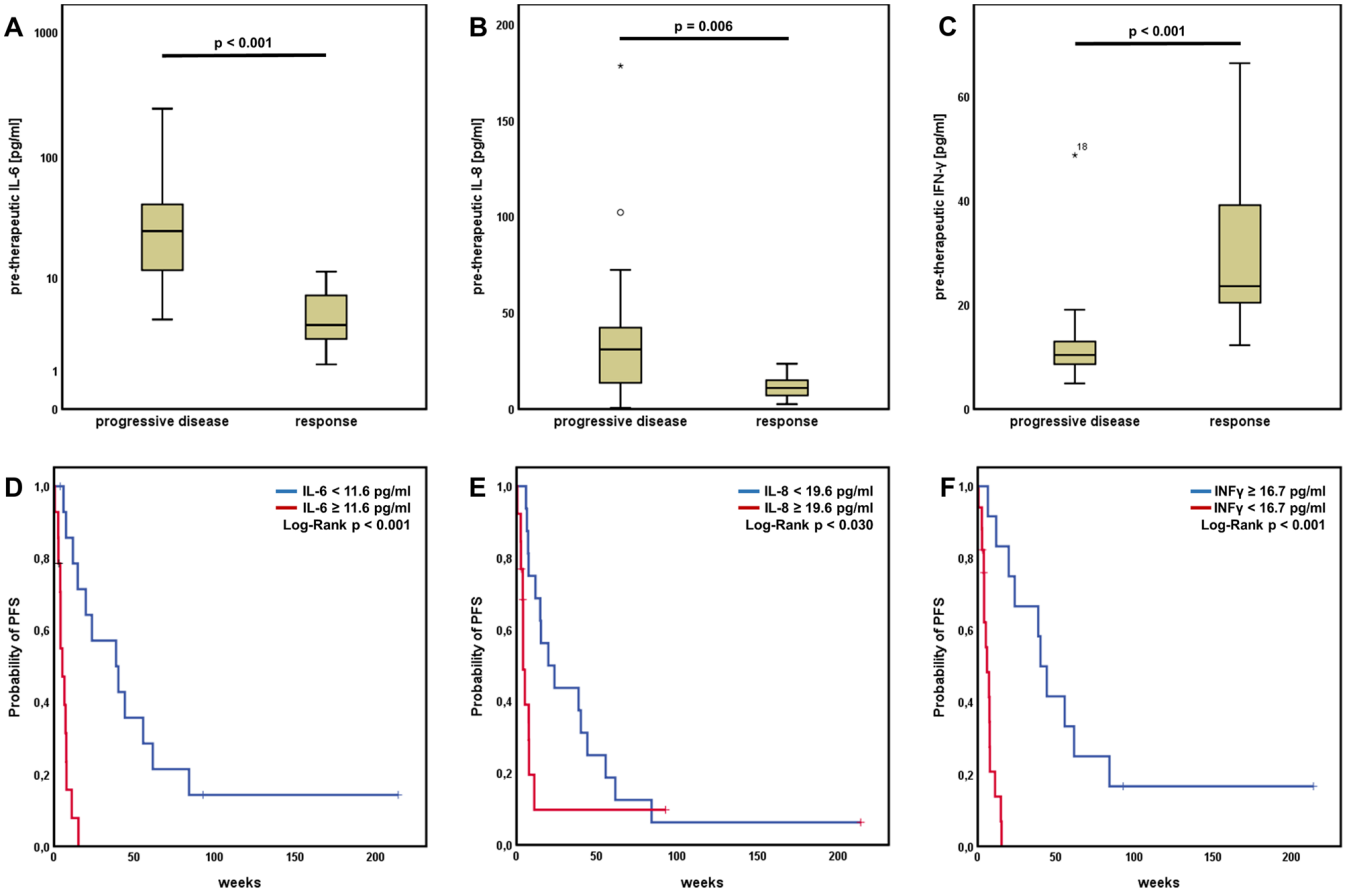
Pre-therapeutic cytokine levels predict response and survival. Patients with initial response showed significantly lower levels of (**A**) interleukin 6 (IL-6), (**B**) interleukin 8 (IL-8) or elevated levels of (**C**) interferon gamma (IFN-γ). PFS was significantly longer in patients with lower IL-6 (**D**), lower IL-8 (**E**) and higher IFN-γ (**F**).

Interleukin-6 (IL-6) was significantly increased in patients showing primary progression under immunotherapy (p < 0.001, Fig. 2A). The best cut-off value for IL-6 regarding response was determined to be 11.6 pg/ml. Patients with IL-6 equal or higher than 11.6 pg/ml had significantly reduced PFS compared with patients harboring lower values (median PFS 5.14 weeks [95% CI 0.95–9.33] vs. 38.57 weeks [95% CI 8.45–68.69], p < 0.001) (Fig. 2D).

Interleukin-8 (IL-8) showed to be significantly elevated in patients with primary progressive disease (p = 0.006, Fig. 2B). 19.6 pg/ml revealed to be the best cutoff for response in our cohort. Patients with IL-8 equal or greater than 19.6 pg/ml showed significantly reduced PFS under checkpoint inhibitor treatment (median PFS 4.0 weeks [95% CI 2.71–5.29] vs. 19.71 weeks [95% CI 3.19–36.23], p = 0.030) (Fig. 2E).

In our cohort, the level of interleukin-10 (IL-10) did not discriminate between response and progressive disease or subgroups with better survival. ROC analysis revealed 19.4 pg/ml as best cut-off value. However, only two patients with very short PFS were allocated into the IL-10 high group so that we did not calculate statistics for the survival curves.

Patients responding to immunotherapy were characterized by significantly increased levels of Interferon-γ (IFN-γ) (p < 0.001, Fig. 2C). An IFN-γ serum level of 16.7 pg/ml was determined as best cut-off in our cohort. Patients with IFN-γ equal or higher than 16.7 pg/ml had significantly improved PFS compared with patients harboring lower values (median PFS 40.0 weeks [95% CI 30.79–49.22] vs. 5.86 weeks [95% CI 2.20–9.51], p < 0.001) (Fig. 2F).

Response to immunotherapy was by trend associated with lower levels of Tumor Necrosis Factor alpha (TNF-α). However, this difference was not statistically significant. Also, with the optimal cut-off of 3.6 pg/ml, there was no statistically significant difference regarding PFS when stratified by TNF-α.

Table 3 summarizes the results of the univariate analyses reported above.

**Table 3:** Results of univariate correlation of inflammation scores and cytokine levels with response and PFS under ICI treatment.

	PD vs. PR	Median PFS
NLR (≥ 5.2 vs. < 5.2)	p = 0.003	Median PFS 7.43 weeks [95% CI 3.37–11.49] vs. 40.0 weeks [95% CI 16.01–64.0], p < 0.001
SII (≥ 1217 vs. < 1217)	p = 0.011	Median PFS 7.51 weeks [95% CI 3.26–11.88] vs. 40.0 weeks [95% CI 11.48–68.52], p = 0.001
PLR (≥ 50 vs. < 50)	p = 0.035	Median PFS 11.71 weeks [95% CI 2.06–13.37] vs. 7.71 weeks [95% CI 0–25.71], p = 0.406
CRP (≥ 4.3 mg/dl vs. < 4.3 mg/dl)	p = 0.67	median PFS 7.57 weeks [95% CI 2.71–12.44] vs. 14.71 weeks [95% CI 4.18–25.25], p = 0.043
mGPS (0 vs. 1 vs. 2)	p = 0.453	Median PFS 15.14 weeks [95% CI 0–34.21] vs. 11.71 weeks [95% CI 2.94–20.49] vs. 7.57 weeks [95% CI NE], p = 0.204
IL-6 (≥ 11.6 pg/ml vs. < 11.6 pg/ml)	p < 0.001	Median PFS 5.14 weeks [95% CI 0.95–9.33] vs. 38.57 weeks [95% CI 8.45–68.69], p < 0.001
IL-8 (≥ 19.67 pg/ml vs. < 19.67 pg/ml)	p = 0.006	Median PFS 4.0 weeks [95% CI 2.71–5.29] vs. 19.71 weeks [95% CI 3.19–36.23], p = 0.030
IL-10 (≥ 19.4 pg/ml vs. < 19.4 pg/ml)	p = 0.842	Median PFS 2.57 weeks [95% CI NE] vs. 11.71 weeks [95% CI 2.86–20.57], p = NE
IFN-γ (≥ 16.7 pg/ml vs. < 16.7 pg/ml)	p < 0.001	Median PFS 40.0 weeks [95% CI 30.79–49.22] vs. 5.86 weeks [95% CI 2.20–9.51], p < 0.001
TNF-α (≥ 3.6 pg/ml vs. < 3.6 pg/ml)	p = 0.387	Median PFS 11.0 weeks [95% CI 5.50–16.50] vs. 7.43 weeks [95% CI 0–19.16], p = 0.207

Integrating the significant biomarkers of the univariate analysis (NLR, SII, PLR, IL-6, IL-8 and IFN-γ) into a multivariate logistic regression model we achieved a predictive value for response of 96.6%.

A multivariate Cox regression revealed IFN-γ as an independent marker for PFS (HR 0.92, 95% CI [0.86–0.97], p = 0.004).

To control the effect of heterogeneity in our cohort, we tested, whether the effects, we found in the total cohort, were also seen in in the subgroups Nivolumab-treatment and Pembrolizumab treatment. By trend, we saw the same results. In some cases, there was no statistical significance due to the small sample size. Importantly, we found that IL-6 was consistently significantly increased in patients with primary progression (Nivolumab-group p = 0.003 and Pembrolizumab-group p = 0.017). Furthermore, IFN-γ was significantly increased in the responder group irrespective of treatment modality (Nivolumab-group p = 0.026 and Pembrolizumab-group p = 0.004). We also analyze, if there are difference regarding the histology. Also in this case we found the same trends and consistent significance of IL-6 (adenocarcinoma-group p = 0.002 and squamous-group p = 0.038) and IFN-γ (adenocarcinoma-group p = 0.003 and squamous-group p = 0.010).

### Inflammatory profile of patients with long-term benefit from single agent immunotherapy

To further characterize patients with long-term benefit from checkpoint inhibition, we compared patients with a PFS of at least 30 weeks to the group with earlier progression. In our cohort 8 patients had long-term response as defined above. This accounts for 27.6% of the total cohort being in line with the results from the pivotal studies showing that about a third of patients have long-term response.

Pre-therapeutic CRP, IL-8, IL-10 and TNFα levels did not significantly differ between the two groups.

**Figure 3:**
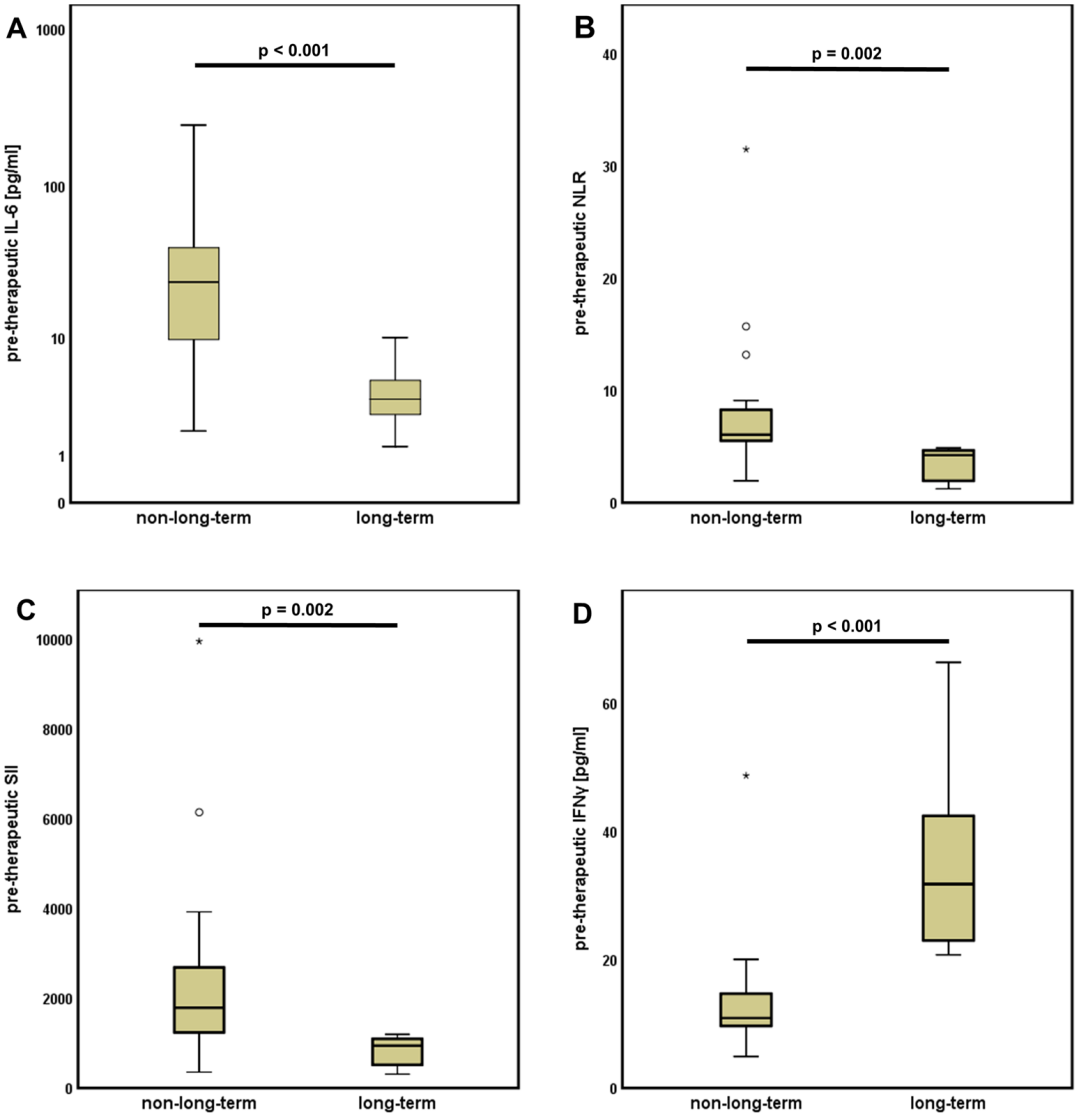
Pre-therapeutic inflammatory scores and cytokine levels predict long-term response to ICI. Patients with long-term response showed significantly lower levels of (**A**) interleukin 6 (IL-6), (**B**) neutrophil to lymphocyte ratio (NLR) and (**C**) systemic inflammation index (SII) or elevated levels of (**D**) interferon gamma (IFN-γ).

However, patients with long-term response showed significantly lower levels of IL-6 (p < 0.001, Fig. 3A), NLR (p = 0.002, Fig. 3B) and SII (p = 0.002, Fig. 3C). Beside this, the long-term responder group was characterized by significantly higher IFNγ values (p < 0.001, Fig. 3D).

**Figure 4:**
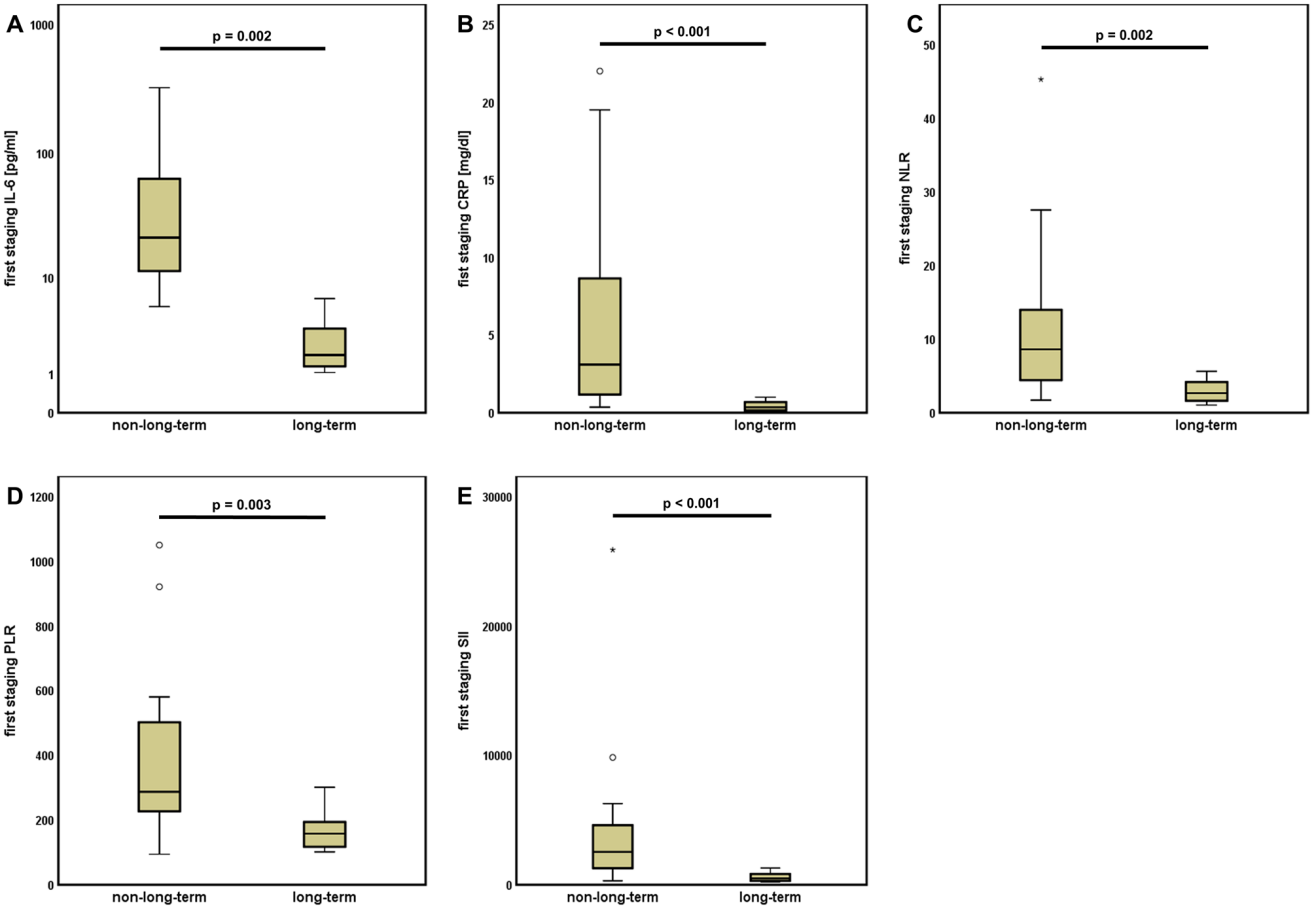
First staging inflammatory scores and cytokine levels predict long-term response to ICI. At the time of the first staging, patients with long-term response showed significantly lower levels of (**A**) interleukin 6 (IL-6), (**B**) CRP and (**C**) neutrophil to lymphocyte ratio (NLR), (**D**) platelet to lymphocyte ratio (PLR) and (**E**) systemic inflammation index (SII).

The results of the pre-therapeutic inflammation profile of long-term responders also translated into the results of the profiles examined at the time of the first response assessment. Significantly lower levels of IL-6 (p < 0.001, Fig. 4A), CRP (p < 0.001, Fig. 4B), NLR (p = 0.002, Fig. 4C), PLR (p = 0.003, Fig. 4D) and SII (p < 0.001, Fig. 4E) characterized the group of patients with long-term response.

### Relevance of inflammatory profile dynamics

**Figure 5:**
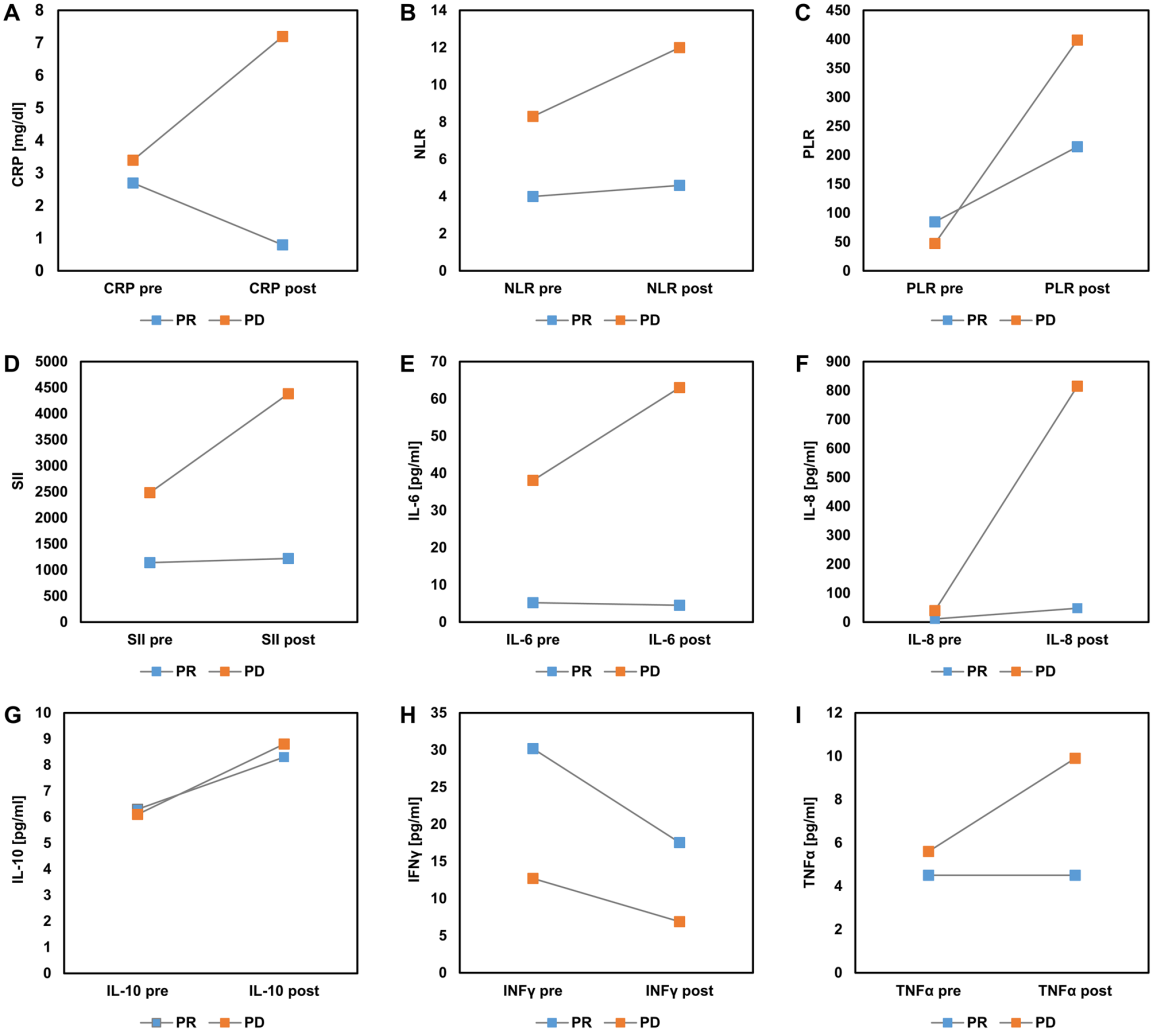
Dynamics of pre-therapeutic to first staging inflammatory scores and cytokine. (**A**) CRP, (**B**) neutrophil to lymphocyte ratio (NLR), (**C**) platelet to lymphocyte ratio (PLR), (**D**) systemic inflammation index (SII), (**E**) interleukin 6 (IL-6), (**F**) interleukin 8 (IL-8), (**G**) interleukin 10 (IL-10), (**H**) interferon gamma (IFN-γ) and (**I**) tumor necrosis factor alpha (TNFα).

In a next step we sought to analyze, whether changes of dynamics of the inflammatory profile over time could indicate which patient would respond to checkpoint inhibition. Figure 5 shows the trend of means (pre-therapeutic and at time of the first staging) divided into primary disease (PD) and response (PR) respectively. We found that all markers indicating an acute systemic inflammation (IL-6, IL-8, TNFα, CRP, NLR, PLR and SII) clearly increased in patients with progressive disease. In contrast, high levels of IFNγ seemed to be a good predictor for response.

In the case of patients with, at least initial response to checkpoint inhibition the trends were not as clear as in the progressing group, indicating that interpretation of dynamics might not be as relevant in this group.

### Assessment of immune related adverse events

Immune related adverse events (irAEs) were monitored during the study period. Altogether, five patients were diagnosed with irAEs (3 hypothyroidisms, 1 exacerbation of a rheumatic arthritis and one light pneumonitis). Table 4 summarizes the detected irAEs as well their severity. No irAE led to discontinuation of treatment. We found no association between appearance of irAEs, response or cytokine levels.

**Table 4:** Detected irAEs in the study cohort.

irAE	Grade of severity (CTCAE)	PD or response group	Discontinuation of treatment
Hypothyroidism	1–2	Response	No
Hypothyroidism	1	Response	No
Hypothyroidism	2	PD	No
Exacerbation of a rheumatic arthritis	2	Response	No
Pneumonitis	1–2	PD	No

*irAE* immune related adverse events.

## Discussion

Here we report the results of a prospective study evaluating the predictive and prognostic potential of pre-therapeutic and longitudinal measurement of inflammation status in NSCLC patients treated with ICIs. Although PD-L 1 expression correlates with response to ICI treatment, it is far from a perfect biomarker. Some patients with high PD-L1 expression do not benefit from ICI and vice versa. Furthermore, only 20–30% of treated patients have long-term response. Therefore, we sought to examine whether inflammatory scores and cytokine profiles can give additional information on patients’ response to this treatment modality.

We found that signs of pre-therapeutic acute inflammation were associated with poor response to ICI treatment and reduced duration of response. Pre-treatment elevated levels of NLR, SII, CRP and PLR revealed to predict poor response and reduced PFS. This is in line with previous studies showing that NLR seems to be a robust predictive marker of response to ICI treatment in several tumor entities^3, 7–10^. 1845 NSCLC patients from 21 studies were analyzed in a comprehensive meta-analysis showing that pre-therapeutic NLR predicts response to ICI treatment^4^. We identified 5.2 to be the best NLR-cut-off in our cohort. This confirms the recommended cut-off of 5 in the above cited meta-analysis^4^. There are also some retrospective data showing a negative prognostic influence of elevated CRP, PLR and SII in different tumor types^3, 11–13^. However, these data are not as conclusive as for NLR and a final cut-off has to be further evaluated.

In our study cohort, we found that patients with primary progression under ICI had significantly increased levels of IL-6 and IL-8, both markers of acute inflammation. This is consistent with other studies showing that both cytokines might be promising biomarkers in NSCLC immunotherapy^6, 14, 15^.

The relevance of acute, IL-6 driven inflammation to response on ICI is also supported by recent basic research showing that mice with high IL-6 expressing tumors have reduced survival when treated with ICI and that blockage of IL-6 reversed the anti PD-L1 resistance^16, 17^.

INFγ stands out against the other markers in our study. We found that increased levels of INFγ were highly predictive of a good and durable response to ICIs. This might be due to the different role of INFγ in inflammation. INFγ stimulates tumor infiltrating lymphocytes (TILs)^6^. Furthermore, INFγ leads to increased PD-L1 expression^18, 19^. Contrary to the other mentioned markers, INFγ induces lymphocyte driven immune response, hereby indicating a synergistic effect with ICI treatment. Other studies also found a positive effect of high INFγ levels on the outcome of ICI treatment^20–22^. However, a clear cut-off for INFγ needs to be evaluated in further research.

A strength of our analysis is the prospective and longitudinal measurement of inflammatory scores and cytokines. Our results show that especially patients not responding to ICI treatment can be identified by increasing levels of pro-inflammatory markers (CRP, NLR, PLR, SII, IL-6, IL-8, TNFα) over time. Therefore, it might be an important approach not to interpret only the sole pre-therapeutic values, but also the dynamics of these markers. This gets support of some recent studies showing that increasing levels of NLR^23, 24^, IL-6^5^ and IL-8^15^ under ICI treatment predict reduced response. On the other hand, increasing levels of INFγ have been found to improve response and survival in NSCLC patients treated with ICI^25^.

Other predictive and prognostic scores beside inflammatory scores have been evaluated to find the best tailored ICI treatment for NSCLC patients. For example, the EPSILoN or the LIPI score combine several baseline characteristics to predict response to ICIs in NSCLC^26, 27^. This illustrates that the search for reliable and sensitive biomarkers in ICI treatment of NSCLC is still a dynamic field.

Our study may be limited in some way. First, we report a small and, in some way, heterogeneous, cohort. The study population is predominantly male cohort. Furthermore, more patients have been treated with nivolumab than with Pembrolizumab. Another limitation might be, that we included squamous as well as non-squamous histologies. However, there are also important strengths. Our analysis is a prospective study and, therefore, stands out against the most data retrospectively evaluating predictive markers in ICI treatment. All patients were initially staged with PET-CT before starting ICI treatment. Therefore, we could exclude different causes explaining difference in cytokine profile or survival by another cause. However, a large independent and prospective validation cohort, would be desirable. Although assessment in second line, as was done for this study, would not be feasible due to a change in treatment patterns during the last years, a confirmation of this could be done prior to first line single agent immunotherapy where pembrolizumab is considered standard of care for most patients with a PD-L1 score of greater than 50%.

Nevertheless, much more research will be needed in the next years to define more precise subgroups to provide a biomarker guided treatment for patients in this setting.

## Conclusion

In conclusion, pre-therapeutic assessment of inflammation and cytokine profiles has the ability to predict response and survival in NSCLC patients treated with single agent ICIs. Especially IL-6 and IFN-γ seem to be promising markers. Furthermore, dynamics of these markers might be a promising tool to characterize long-term responder.
